# Relation of life sciences students’ metacognitive monitoring to neural activity during biology error detection

**DOI:** 10.1038/s41539-024-00231-z

**Published:** 2024-03-04

**Authors:** Mei Grace Behrendt, Carrie Clark, McKenna Elliott, Joseph Dauer

**Affiliations:** 1 Department of Educational Psychology, University of Lincoln-Nebraska, Lincoln, NE USA; 2 School of Natural Resources, University of Nebraska-Lincoln, Lincoln, NE USA

**Keywords:** Human behaviour, Neuroscience

## Abstract

Metacognitive calibration—the capacity to accurately self-assess one’s performance—forms the basis for error detection and self-monitoring and is a potential catalyst for conceptual change. Limited brain imaging research on authentic learning tasks implicates the lateral prefrontal and anterior cingulate brain regions in expert scientific reasoning. This study aimed to determine how variation in undergraduate life sciences students’ metacognitive calibration relates to their brain activity when evaluating the accuracy of biological models. Fifty undergraduate students enrolled in an introductory life sciences course completed a biology model error detection task during fMRI. Students with higher metacognitive calibration recruited lateral prefrontal regions linked in prior research to expert STEM reasoning to a greater extent than those with lower metacognitive calibration. Findings suggest that metacognition relates to important individual differences in undergraduate students’ use of neural resources during an authentic educational task and underscore the importance of fostering metacognitive calibration in the classroom.

## Introduction

Self-regulated learning is the extent to which students are engaged, motivated, and behaviorally active in their learning^1^. Self-regulated learners accomplish tasks or goals by continuously monitoring and correcting the effectiveness of their strategies and past behaviors to better control future learning^2^. Metacognitive calibration, the match between a student’s objective performance and their subjective self-assessment of that performance^3,4^, is fundamental to self-regulated learning because it forms the basis for performance monitoring^5^. Students with more accurate calibration presumably have more reliable information on which to base their deployment of effortful, strategic, cognitive resources to optimize task performance^6^. Metacognitive calibration has been linked to dorsomedial prefrontal systems thought to be involved in monitoring and detecting errors in performance, as well as to lateral PFC regions linked to effortful cognitive control^7^. However, neuroimaging studies of metacognitive calibration generally have used experimental tasks rather than tasks reflective of authentic educational experiences, leaving a gap in understanding how metacognition relates to students’ neural processes in classroom-relevant learning. Modeling and model evaluation are authentic tasks in scientific practice and education^8^, and expert scientists are skilled at detecting inaccuracies in models^9^. This study aimed to determine the relation of metacognitive calibration to student performance and neural activity when evaluating biological models.

Although a general definition of metacognition is “thinking about thinking”^10^, contemporary conceptualizations typically divide it into two distinct processes: knowledge about cognition, including self-monitoring, and self-regulatory mechanisms^10–12^. Self-monitoring involves knowing how well one is performing and recognizing the likelihood of accuracy or inaccuracy in one’s judgments or behaviors. Conversely, metacognitive self-regulation concerns the process of organizing one’s cognition, planning, being aware of one’s comprehension, and evaluating the efficacy of strategies during task performance^13,14^. Learners who develop high self-monitoring and self-regulation skills revise and reconstruct concepts to advance their knowledge and achieve high self-efficacy, persistence, and self-discipline in performing tasks^15–17^. Well-calibrated self-assessments also have been linked to students’ use of more effective study strategies and to higher levels of academic growth over time^18–20^.

Student ratings of confidence in their own performance offer one means of assessing self-awareness and calibration^21^. For well-calibrated learners, confidence ratings should correspond with actual performance^22^. That is, students should have high confidence when they are accurate and low confidence when not. Although confidence estimates are higher for correct relative to incorrect trials^23^, lower-performing students tend to be more overconfident in their performance predictions, while higher-performing learners are accurate or underconfident in their self-assessments^12,24,25^. Additionally, learners tend to be overconfident when evaluating complex or difficult information and underconfident about relatively easier information^26^.

Metacognition has repeatedly been linked to the prefrontal cortex (PFC), with greater activity in the dorsolateral and anterior medial PFC being associated with higher levels of self-awareness and metacognitive accuracy^27–29^. Several metacognition studies highlight the ventromedial PFC and posterior medial frontal cortex as central brain areas related to confidence estimates^30,31^ and error detection processes^32–34^, while both the frontopolar cortex and the lateral PFC are involved in explicit metacognitive judgments^23,35^, metacognitive control, and subsequent behavioral regulation^36,37^. More generally, areas in the dorsal anterior cingulate cortex (ACC) are thought to monitor for conflict or errors in performance, whereas the lateral PFC presumably uses these inputs to bias behavior toward more adaptive cognitive strategies^7,35,38^. From these studies, it is reasonable to hypothesize that students with higher levels of metacognitive calibration will show higher levels of activity in medial and lateral PFC regions during academic tasks, reflecting optimal use of self-monitoring and regulatory processes to guide performance. However, given that these studies have often involved experimental tasks, the extent to which these same neural regions are linked to metacognition during more authentic educational tasks remains unclear.

Model-based reasoning, a core emphasis area in STEM education, offers a particularly relevant context for understanding how metacognitive calibration relates to students’ brain activity during authentic learning experiences^39^. From simple flowcharts and graphs to complex computer simulations, models pervade all areas of science and are how scientists reason, evaluate hypotheses, and convey ideas^40,41^. Undergraduate biology students encounter models through textbooks, lectures, note-taking, and classroom activities that convey foundational introductory principles regarding genetic, ecological, and cellular systems^42^. Each time they encounter a model, students must determine whether their prior knowledge of the topic is in agreement with the observed model. Measuring biology students’ capacity to detect errors in models constitutes a powerful means to assess the relation of their metacognition to their performance of this critical scientific skill.

Linking metacognitive calibration to model error detection also offers the potential for understanding how to elicit conceptual change to ultimately foster a deeper understanding of fundamental scientific concepts^43^. To learn scientific concepts, students must be able to actively refine their learning process and integrate new concepts and relationships with their prior knowledge^44^. Learners must first become dissatisfied with their existing conceptions, embracing new conceptions as plausible^45^. However, conceptual alterations prove difficult when learners hold misconceptions^46,47^ because these errors continue to be recalled and reinforced until scientifically accurate knowledge outcompetes them^48^. Absent or poor metacognition may predict the extent to which learners both ignore new information and resist changing their minds, even when new information indicates errors in their original beliefs^27^. For learners who hold misconceptions, conceptual change is more predictable when an alternative scientific viewpoint initiates cognitive conflict, allowing error detection in the misconception^49^.

Metacognitive calibration may be a critical initial step in the process of conceptual change^50^. Students who are more metacognitively aware of misconceptions about a given topic are more likely to detect and remedy those misconceptions to develop a deeper understanding of a concept or topic, decreasing the likelihood of entrenching misconceptions^51^. Conversely, students who are unaware of their misconceptions may become overly secure in those misconceptions (i.e., they do not know what they do not know)^51,52^ and may consequently miss opportunities for knowledge revision^53^. Evaluating how students’ metacognitive calibration links to their error detection and ultimate learning may offer clues as to how metacognition supports conceptual change.

Limited literature on the neural regions involved in expert science cognition highlights its association with prefrontal regions linked to error detection, conflict monitoring, and inhibition^54^. Additionally, experts evaluating the accuracy of scientific circuits activate anterior cingulate cortex (ACC), ventrolateral PFC (VLPFC), and dorsolateral PFC (DLPFC) to a greater extent than novices, who tend to activate more limited prefrontal regions^55,56^. These brain regions are thought to work in concert to support scientific reasoning. The lateral PFC provides the cognitive control necessary for logical thinking, flexible reasoning, and inhibition of inappropriate responses or attentional stimuli^32,57^; the ventromedial PFC integrates emotional and cognitive factors to guide decision-making^56,58^; and the ACC contributes to inhibitory processes and error monitoring, helping individuals stay on track and correct or adjust their reasoning when necessary^59^.

With respect to metacognition, novice students exhibited greater dorsomedial frontal activity when they were confident vs. not confident in their responses to physics electric circuit diagrams^60^. Taken together, these studies implicate ACC and lateral PFC regions in the expert processing and resolution of science misconceptions. Assuming that students with higher metacognitive calibration are better equipped to engage in ‘expert’-like error detection and associated engagement of control, we expected such students to use these regions to a greater extent than their less calibrated peers when evaluating biological models.

Conceptual change theories identify metacognitive calibration as a critical process that enables students to detect and resolve gaps in their knowledge. However, neuroimaging studies typically examine the neural bases of metacognitive processing and calibration accuracy in the context of novel or self-reflection-based tasks, rather than educationally authentic tasks like those involving scientific reasoning^61,62^. Further understanding of the neural bases of metacognitive monitoring during an authentic task of model error detection will offer new insights into the mechanisms by which this construct supports students’ development as scientists. The limited neuroimaging research on scientific cognition indicates that ‘expert’ scientists activate DLPFC, VLPFC, and ACC brain regions to a greater extent than ‘novice’ undergraduates when reasoning about scientific phenomena, particularly when accurate reasoning involves detecting and inhibiting misconception^55,63^. The current study’s goal was to examine the relation of students’ metacognitive calibration to their neural activity during a biological model evaluation task. Specifically, we aimed to determine how individual differences in students’ calibration accuracy^64,65^ related to their activity in lateral prefrontal and ACC regions linked both to metacognition and to scientific expertise (see “Methods” section). Based on the theory that accurate calibration serves as a foundation for error detection and conceptual change, we hypothesized that students with higher metacognitive calibration would show higher levels of activity in prefrontal regions linked to these scientific processes.

## Results

### Behavioral results

**Fig. 1:**
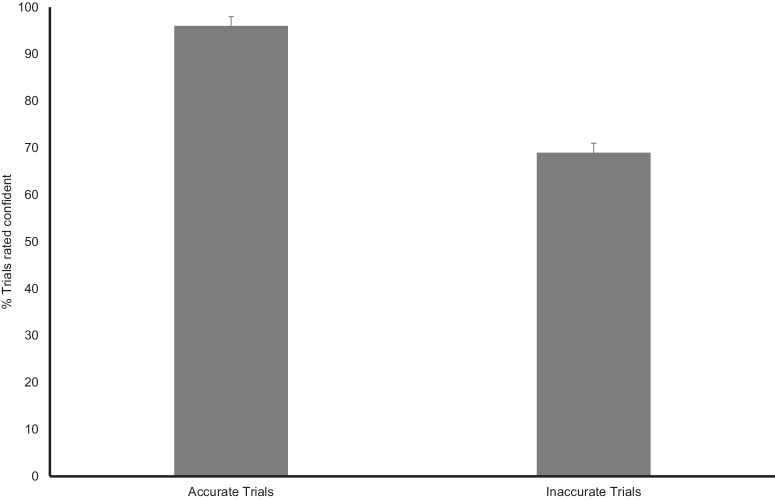
Student confidence levels for accurate vs. inaccurate trials in the model evaluation task. Proportion of accurate and inaccurate trials for which students responded that they were confident during the model evaluation task. *Note*: Error bars = standard error of the mean.

Table 1 presents descriptive statistics and correlations among the different behavioral variables. Students on average were correct on 65% of the model evaluation task trials, whereas they were confident ~75% of the time. Figure 1 illustrates the significant difference in student confidence levels for accurate vs. inaccurate trials of the model evaluation task (*t*(49) = 7.72, *p* < 0.001, *d* = 1.09), although students still responded that they were confident more often than not for trials where they made an inaccurate response. The moderate correlation between total accurate responses and total confident responses (*r* = 0.403, *p* = 0.004) indicated that as participants’ accuracy increased, their overall confidence levels also increased (for further behavioral findings highlighting the importance of self-monitoring sees^1,2^).

**Table 1:** Descriptive statistics and correlations among the behavioral variables

Variable *M* (SD)	1	2	3	4	5	6	7	8	9	10
1. Total accuracy 23.40 (4.02)										
2. Total responses of ‘confident’ 27.28 (4.38)	0.40**									
3. Phi^a^ 0.10 (0.19)	0.25	0.17								
4. Confidence bias −0.27 (0.25)	0.16	0.39**	−0.84**							
5. Type 1 d’ 0.90 (0.67)	0.97**	0.36**	0.28	0.09						
6. Meta *d*’ 0.72 (0.89)	0.54**	0.30*	0.90**	−0.61**	0.57**					
7. Meta-efficiency −0.18 (0.75)	−0.23	0.03	0.82**	−0.81**	−0.22	0.67**				
8. Biology self-efficacy 3.78 (0.53)	0.23	0.24	0.11	0.07	0.20	0.18	0.03			
9. KBIT Reading 106.36 (9.28)	0.13	−0.01	0.06	0.02	0.16	0.07	−0.06	0.03		
10. Engagement 4 (0.47)	−0.02	−0.09	−0.12	0.08	−0.06	−0.18	−0.16	0.12	−0.01	
11. Final Course Grade 3.55 (0.62)	0.40**	−0.13	0.23	−0.13	0.34*	0.28*	0.03	0.21	0.19	0.23

*p < 0.05, **p < 0.01.

^a^*N* = 47.

The different metrics for metacognitive calibration, sensitivity, and efficiency, as calculated from students’ confidence ratings and accuracy in the fMRI model evaluation task, were robustly inter-correlated (*p*’s < 0.01). Neither student’s ϕ scores nor their metacognitive efficiency scores correlated with their model evaluation accuracy, although meta-*d*’ scores did, as expected (*p* < 0.001). Final course grade in the introductory life science course correlated slightly with model evaluation task accuracy (*p* = 0.008), but not with confidence, *p* = 0.39. Measures of metacognition showed minimal correlations with final course grades, except for meta-*d’*, *p* = 0.05.

### Neuroimaging results from the model evaluation task

#### Overall patterns of brain activity for confident and non-confident responses

**Fig. 2:**
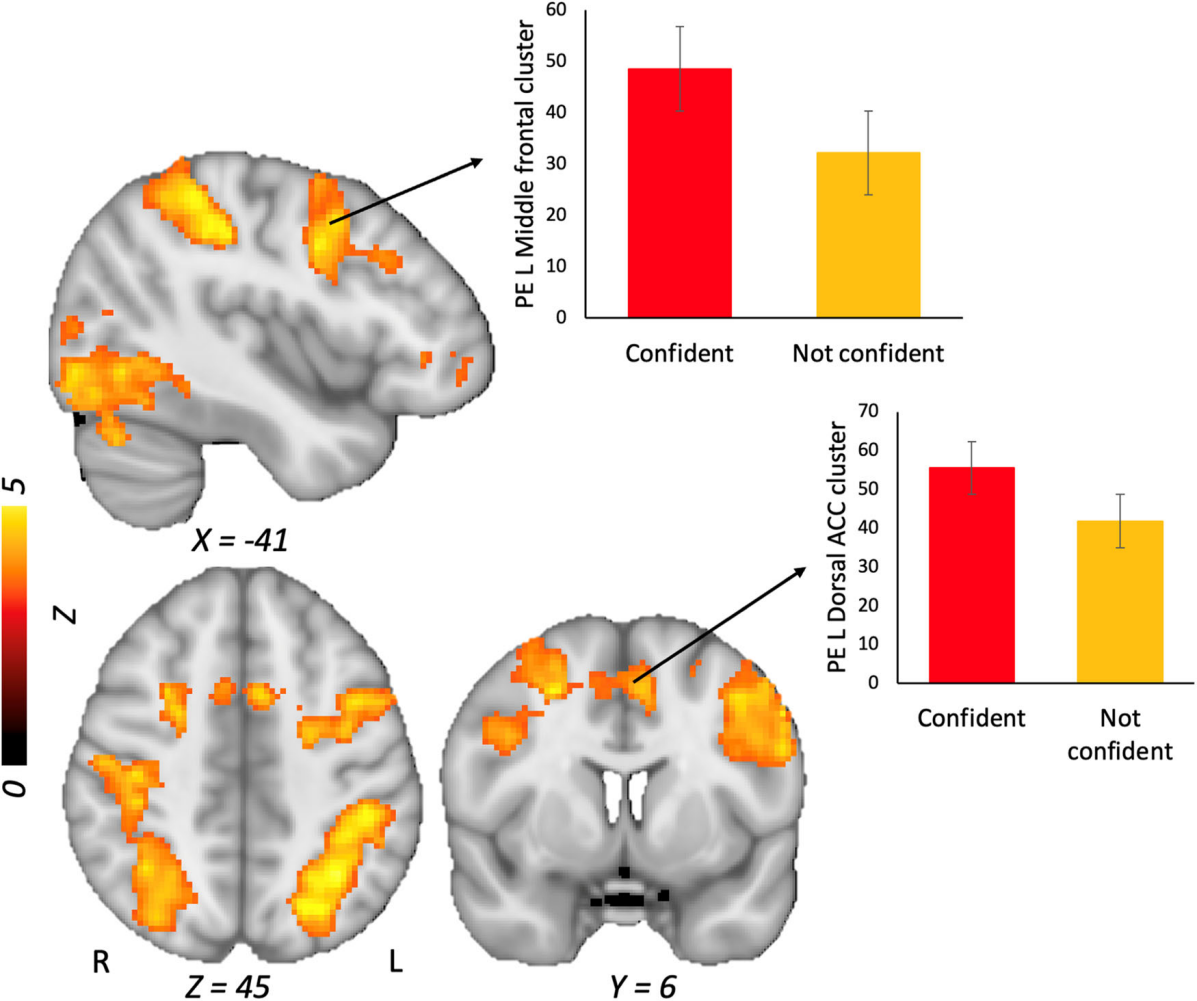
Contrast of confident > non-confident trials in the model evaluation task. *Note:* PE parameter estimate. Graphs reflect the mean parameter estimates extracted from the cluster. Error bars reflect standard errors of the mean.

Table 2 and Fig. 2 show that during trials where students indicated they were Confident > Not confident, widespread neural activation occurred across occipital regions, and in the parietal cortex and lateral and medial PFC (see Supplementary materials for the same analysis using a more conservative statistical threshold). We performed a further contrast of Error > No error models only for trials where students were confident, which indicated that students showed increased activity in a medial frontal cluster, in bilateral inferior prefrontal/insular regions, and in the lingual gyrus (Table 2, Supplementary Fig. 2). Finally, we contrasted trials where students were confident and accurate in their response > confident but inaccurate (i.e., overconfident) in their response. This analysis revealed that students exhibited more activity in a single cluster in the left lingual gyrus when they were confident and accurate relative to confident and inaccurate (Table 2, Supplementary Fig. 3). Overall when examined by trial type, students exhibited greater activity in lateral and medial PFC regions when they were confident, although PFC activity did not diverge for trials where students were overconfident vs. calibrated in their responses.

**Table 2:** Maximum coordinates for neural clusters with significant BOLD signal change for different trial type contrasts

Contrast	Voxels	Max *Z*	*p*	MNI			Brain region
				*x*	*y*	*z*	
Confident > Not confident	22,248	5.7	<0.001	−34	−58	48	L Inferior parietal lobule (BA 7)
	2312	5.2	<0.001	−56	10	28	L Inferior frontal gyrus (BA 9)
	383	4.63	<0.001	−4	10	50	Medial frontal gyrus (BA 6)
	272	4.51	<0.001	30	22	8	R Insula
	239	4.52	<0.001	20	40	−10	R Anterior cingulate (BA 10)
	230	4.69	<0.001	12	−18	10	R Thalamus
	188	4.56	0.001	26	−42	−16	R Parahippocampal gyrus (BA 37)
	183	4.19	0.002	−32	36	4	Inferior frontal gyrus (BA 47)
	120	4.5	0.02	−34	−20	−10	L Hippocampus
	98	4.34	0.040	−10	−36	−18	Cerebellum
Error model confident > No error model confident	201	4.68	<0.001	−6	48	6	L. Anterior cingulate (BA 32)
	165	3.84	0.002	−12	−80	2	L. Lingual gyrus (BA 17)
	142	4.49	0.005	−32	20	−16	L. Inferior frontal gyrus (BA 47)
	106	4.44	0.023	44	22	−18	R. Inferior frontal gyrus (BA 47)
Confident accurate > Confident inaccurate response	333	3.93	<0.001	−6	−84	−14	L. Lingual gyrus (BA 17)

### Relation of individual differences in student confidence to neural response patterns

**Fig. 3:**
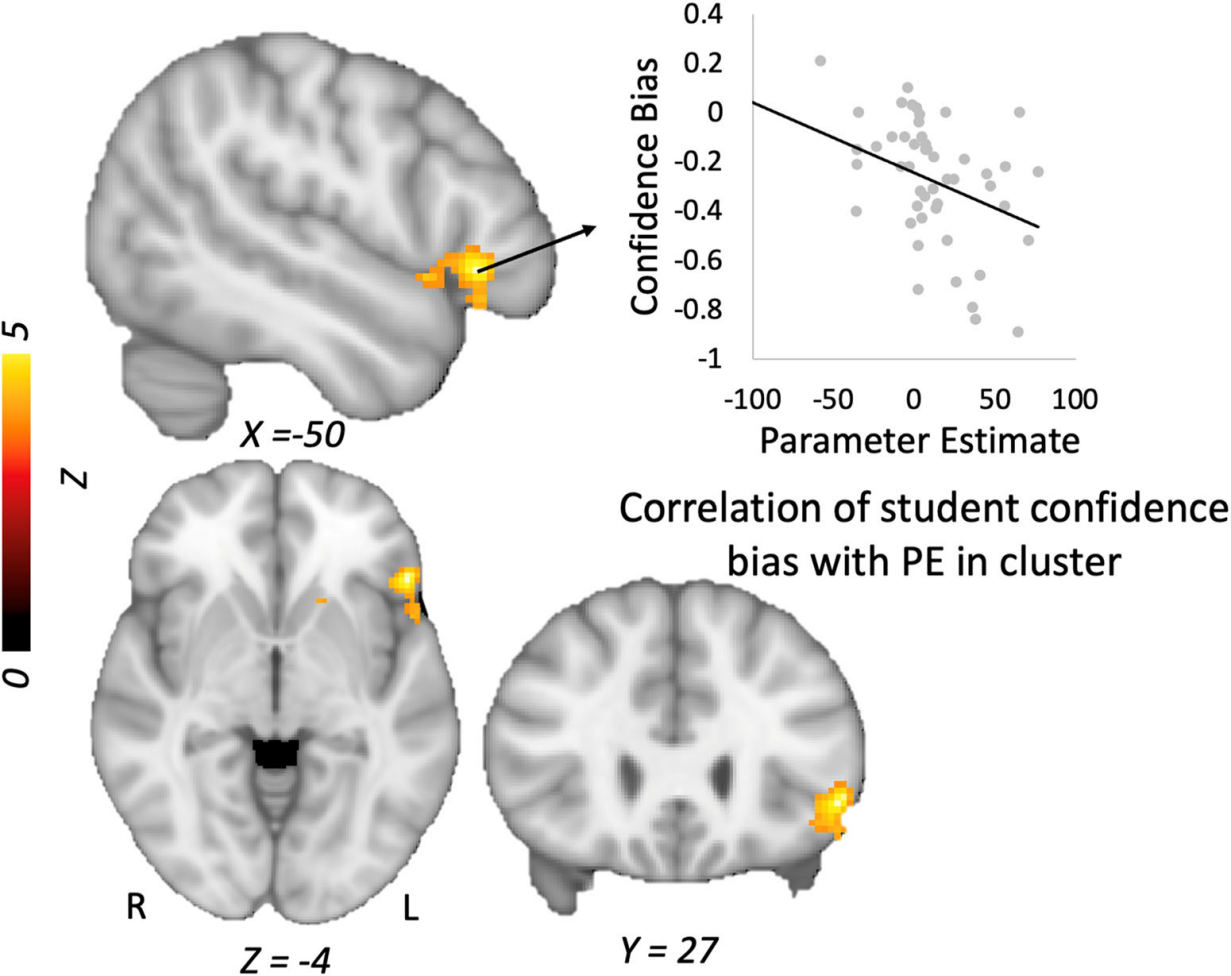
Confident and accurate relative to confident and inaccurate trials in the model evaluation task. Relation of confidence bias to neural activity in left inferior prefrontal cortex during students’ evaluation of error > no error models. *Note:* Graph is shown for illustrative purposes and reflects mean parameter estimates extracted from the cluster. PE Parameter estimate.

Focusing on the Error > No error model contrast, we evaluated the relation of overall accuracy and confidence to students’ neural responses by including these variables as covariates of interest in the group design matrix. There was no association of students’ model evaluation accuracy with their neural response patterns for this contrast. Likewise, students’ overall confidence was not linked to their brain activity. However, students with lower confidence bias showed more activity in two clusters centered in the left inferior frontal gyrus (Table 3, Fig. 3).

**Table 3:** Maximum coordinates for correlations of student metacognitive metrics with BOLD activity during model error detection

Variable	Voxels	Max *Z*	*p*	*x*	*y*	*z*	Brain region
Confidence bias	265	4.3	<0.001	−50	26	−4	L Inferior frontal gyrus (BA 47)
	129	4.35	0.007	−4	16	6	L. Inferior frontal gyrus (BA 44)
Phi	149	4.25	0.003	40	44	22	R. Middle frontal gyrus (B9)
Meta-*d*'	128	4.11	0.007	−46	−56	46	L. Inf parietal lobule (BA 40)
	100	4.53	0.002	−24	−28	68	Postcentral gyrus (BA 3)
	91	3.88	0.011	−32	−80	38	Superior occipital gyrus (BA 19)
Meta-efficiency	105	4.31	0.022	−24	−28	68	L. Postcentral gyrus (BA 3)
	89	4.09	0.045	40	42	20	R. Middle frontal gyrus (BA 9)

All correlations are with the contrast of Model Error > No Error; correlations with phi and confidence bias control for students’ error detection accuracy.

### Relation of student metacognitive calibration to neural response patterns

**Fig. 4:**
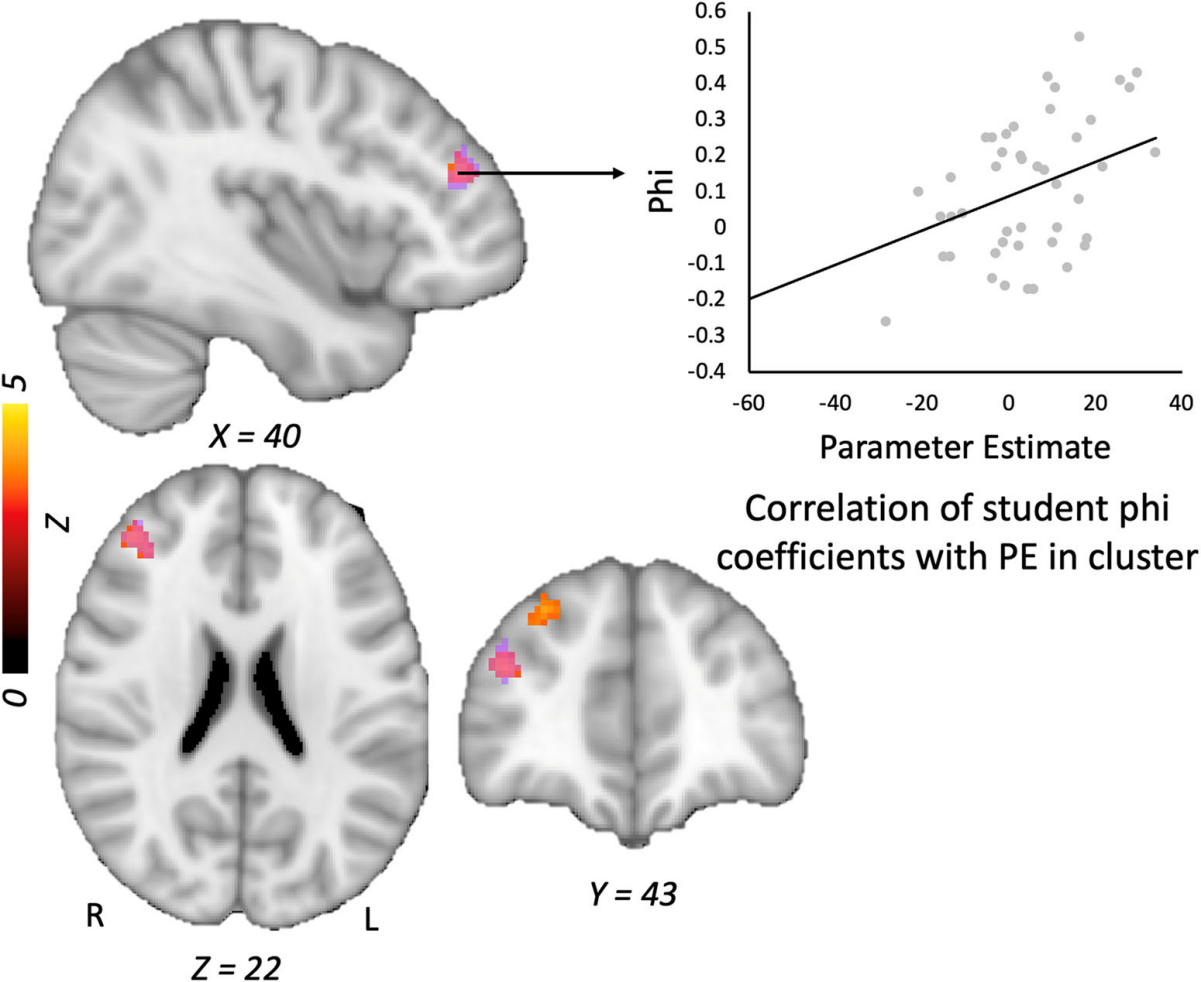
Phi correlation and students’ metacognitive efficiency. Relation of student ϕ (orange) and metacognitive efficiency (purple) sores with activity in the right middle frontal gyrus during students’ evaluation of error > no error models. *Note:* Graph is shown for illustrative purposes and reflects mean parameter estimates extracted from the cluster. PE parameter estimate.

**Fig. 5:**
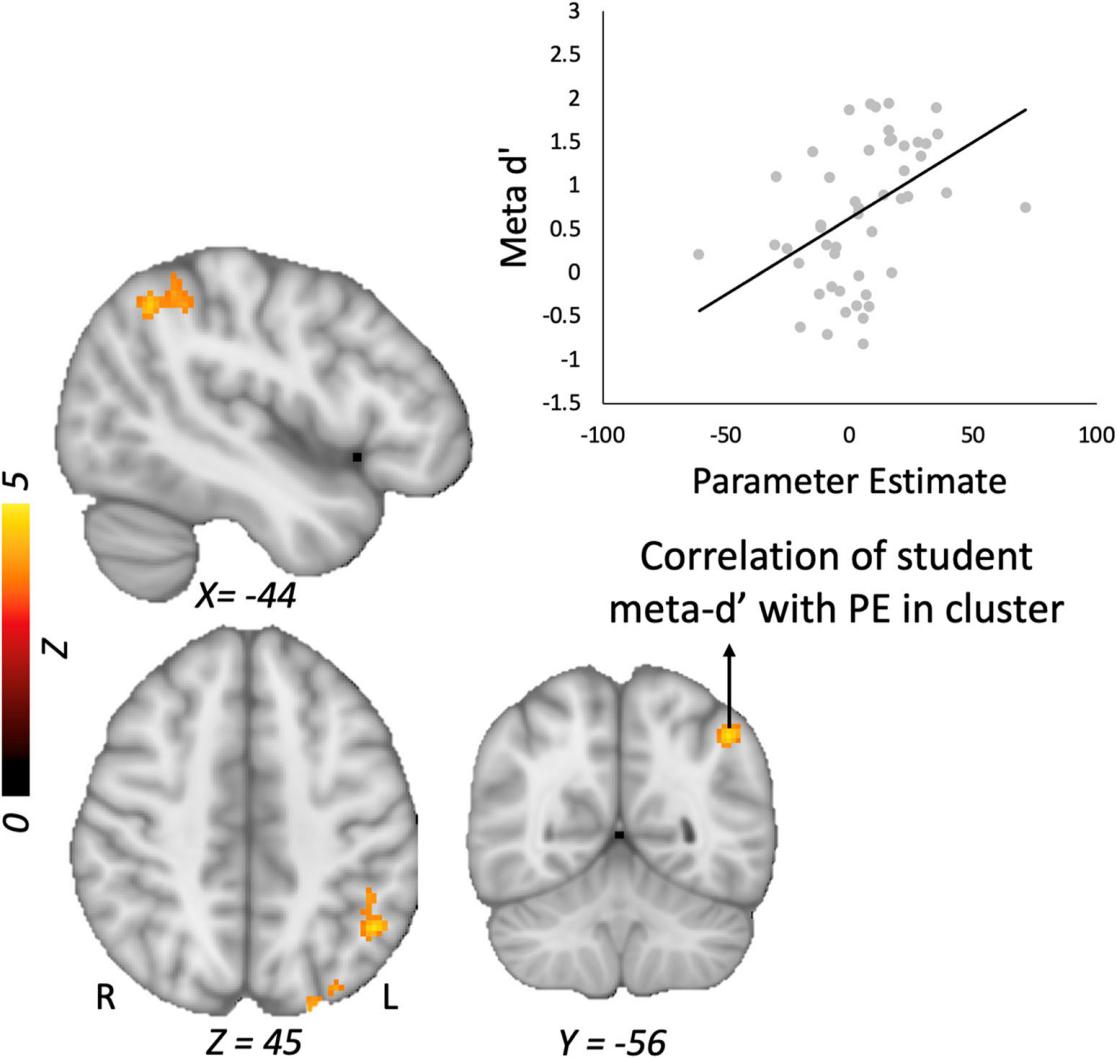
Students’ meta-*d*’ scores and error > no error contrast. Correlation of student *meta-d’* scores with left inferior parietal activity during the evaluation of error > no error models. *Note:* Graph is shown for illustrative purposes and reflects mean parameter estimates extracted from the cluster.

Controlling for accuracy on the model evaluation task, students’ ϕ values were positively associated with and their level of activity in the right middle frontal gyrus for the Error > No error model contrast (see Table 3). Students with higher ϕ scores showed a greater increase in activity in this cluster, which spanned BA 8 and 9 (Fig. 4). Comparable results emerged when we instead used the metacognitive efficiency score (i.e., *d*’–meta *d*’), which was positively linked to activation in a right middle frontal cluster that overlapped with the cluster identified for ϕ. Controlling for accuracy, students’ meta-*d’* scores were associated with activity in the left inferior parietal, superior occipital and postcentral gyri (Fig. 5).

## Discussion

Metacognition is central to science education: it is critical to conceptual change and to cultivating a deep understanding of scientific concepts^66^. Specifically, in biology education, students with a higher awareness of the learning process and a stronger ability to monitor, regulate, and control learning manifest a more meaningful understanding of targeted biology concepts and better scientific inquiry skills^67,68^. The current study evaluated the relation of students’ metacognitive calibration to their neural activity when evaluating errors in biology models. A major finding is that students with higher metacognitive calibration and efficiency recruit lateral prefrontal regions to a greater extent than their peers with lower metacognitive calibration when evaluating error-containing models. This finding has implications for understanding the role of metacognitive monitoring in students’ learning behavior and for approaches to STEM instruction.

Controlling for task accuracy, higher metacognitive calibration, as measured using phi, was linked to higher levels of activity in the right dorsolateral PFC. The same effect was when we instead used students’ metacognitive efficiency scores, which more rigorously parse metacognitive calibration from confidence response biases and task performance^65^. Those few neuroimaging studies that have focused on STEM learning have linked the recruitment of lateral prefrontal and ACC brain regions to ‘expert’ scientific reasoning, with the lateral PFC being especially linked to accurate error detection within STEM experts’ domains of expertise^9,59^. Therefore, the students in our study with higher levels of calibration may be more ‘expert-like’ in the regions they deploy when evaluating error-containing models. Brault Foisy and colleagues^63^ suggested that lateral prefrontal activity may be especially relevant for resolving interference when viewing scientific errors or misconceptions. Given that the DLPFC forms a core part of the fronto-parietal network associated with executive control^69^, findings may indicate that these students are more effectively deploying executive resources to resolve the errors present in these models. Against a growing literature showing that error detection and the associated recruitment of lateral PFC is a marker of STEM expertise, our study links the lateral PFC recruitment to students’ metacognitive calibration. Effective metacognitive calibration may act as a bridge to conceptual change by facilitating students’ use of higher-level conflict resolution and executive resources associated with the lateral PFC.

Surprisingly, students’ behavioral phi, meta-efficiency, and confidence bias scores were not linked significantly to their ultimate course grades, although both model evaluation accuracy and meta-*d’* scores were significantly correlated with course grades. Meta-*d*’ reflects the predicted *d’* of an individual, given their proportions of calibrated vs. non-calibrated confidence ratings^70^. The more meaningful indicator of a student’s metacognitive calibration relative to other students is thus the metacognitive efficiency score. The lack of correlation between meta-efficiency and course grades may indicate that, once we consider the individual’s error detection performance, calibration is less related to overall class grades. Thus, although higher calibration may be tied to the use of prefrontal brain regions associated with effortful resource deployment and, in previous studies, to students’ self-regulated learning and academic success^71–73^, it is students’ mastery of the concepts in these models and their ability to recognize accurate relative to inaccurate models that ultimately is reflected in their course performance. In summary, we discovered that the neural results were distinct from our behavioral results, indicating that there may be neural implications of metacognitive calibration that cannot be inferred only from behavioral results.

Students’ mean confidence was slightly higher than their accuracy (27 vs. 23) on the model task and students in general responded that they were confident more often than not for inaccurate trials. Indeed, some students had a biased score that exceeded 0, which means that they had more confident, inaccurate trials than confident, accurate trials. These descriptive findings support the notion that students tend to overestimate their performance, particularly when performance is low^25^. This overconfidence in one’s abilities suggests a lack of metacognitive awareness of their deficits, which may lead to ineffective self-regulation skills^12^. For example, an overconfident student, believing they know the material, might decide not to study for a test, thus increasing that student’s probability of doing poorly due to inadequate preparation.

Although we did not find an association between confidence bias and performance or course grades, we did find that confidence bias correlated with lower metacognitive efficiency and with less left inferior frontal signal change when students viewed error-containing models. Bellon et al.^22^ found that children showed activation in the left inferior frontal gyrus when rating their confidence in arithmetic problem-solving and that the extent of children’s activity in this region was linked to their mathematics performance. Unlike Bellon et al., we examined neural activity during model evaluation as opposed to during the interval when students were making confidence ratings. However, the overlapping link to the left inferior frontal gyrus perhaps suggests that this region is important for modulating links between confidence and achievement.

Interestingly, differences in neural activity for accurate and confident relative to inaccurate and confident trials were confined to the lingual gyrus; there were no differences in prefrontal activity for this contrast. Potvin et al.^60^ similarly found that calibrated vs. overconfident trials in an electric circuit validation task were linked to parietal, premotor, and fusiform regions rather than prefrontal regions. It is possible that this activity in posterior regions for correct and calibrated trials reflects the use of visual processing resources to support accurate processing and evaluation of the model.

A significant study limitation is that one model evaluation task is not necessarily synonymous with overall academic performance or general metacognitive abilities. Additional assessments would be beneficial for determining and evaluating the neural and behavioral effects of metacognitive processes and of different forms of instruction. It is also important to note that the relationship between brain activity and metacognitive calibration is complex and multifaceted. Different aspects of metacognition, such as monitoring, control, and evaluation, may involve distinct brain regions and networks. Individual differences in brain structure and functioning, as well as task-specific factors, can influence the relationship between brain activity and metacognition. There is continued debate, for example, as to whether metacognitive calibration is task-specific or a general, trait-like characteristic^74,75^.

Another limitation of the study was that we had insufficient trial numbers to fully cross accuracy and confidence at the trial level to examine implications for brain activity: high levels of confidence meant that there were small numbers of trials where students were underconfident. Contrasts of confident vs. non-confident trials or calibrated vs. overconfident trials may also be less reliable because of unbalanced trial numbers for these conditions. Moreover, given the complexity of the task stimuli, each error-containing model was equated in complexity to non-error-containing models. Because the control for stimulus complexity was lost when we removed inaccurate trials, our primary contrast was based on all trials, regardless of students’ accuracy. Future studies should examine how accuracy modulates these relationships. Other limitations included a disproportionate representation of females and Euro-American Whites, as well as a small range in GPAs, which may have limited our power to capture the full extent of variation in students’ metacognitive monitoring performance.

Future research is needed to determine how these individual differences in brain activity relate to ongoing academic achievement. One especially intriguing question is whether and how manipulations to instructional design, such as immediate feedback following errors, may impact students' brain activity and, ultimately, help students become more self-regulated learners^76^. Likewise, future research should also examine how students might be encouraged to reflect on their calibration accuracy and whether such reflection might aid in the process of conceptual change in science. Developing a more nuanced confidence scale might be beneficial to examine confidence judgments in relation to metacognitive calibration. Lastly, additional research is needed to determine how metacognitive calibration affects students’ learning and academic performance throughout their educational trajectory to understand learning acquisition for longer-term retention.

The present study’s finding that students with higher metacognitive monitoring and less confidence bias deployed more ‘expert-like’ lateral prefrontal activity when they encountered error-containing models underscores a need to foster and nurture metacognition and self-awareness in the classroom. Conceptual change is theorized to drive science learning and hinges on students’ willingness to engage with or experience conflict from new ideas^45,47,55,77–79^. Error detection is crucial for effective learning, helping students to identify knowledge gaps, facilitating deeper processing and reflection, and ultimately leading to improved mastery. Embracing errors as valuable learning opportunities can significantly enhance the learning process and foster continuous growth and improvement^80–82^. Students who persistently learn from their own errors through self-reflection may be more motivated to continue to learn after failure, to correct their errors, and to recognize misconceptions^83,84^. Instructors might provide opportunities for students to reflect on their coursework by focusing on the learning process rather than on the content itself^85^, thereby engaging students in reflexive and adaptive thinking. They may also provide direct and immediate feedback, a core mechanism of self-regulated learning, which can positively impact academic achievement^86^. Finally, instructors may need to teach effective study habits to help students act on their metacognitive judgements, as students may lack this knowledge even when they are prepared to alter their strategies^87^. These instructional strategies may represent effective pathways for helping STEM students to be active, self-regulated learners and for moving them toward more expert model evaluation capacities.

## Method

### Participants

Fifty-one undergraduate students were recruited through class announcements from five separate sections of one introductory life sciences course taught by two different instructors in two consecutive academic years at a large Midwestern university. First, we recruited from the sections taught by a professor who heavily used model-based instruction (*n* = 35 students). To maximize our sample size from the following academic year’s sections we recruited more students from one section taught by the professor who also used model-based instruction (*n* = 6), and students from sections of the same course taught by an instructor who used less model-based instruction (*n* = 10). Both instructors have taught the introductory life sciences course for over nine years and used models regularly in their pedagogy. While specific models in this study were never seen or used in the course, the concepts are foundational to biology. While students may have been exposed to textbooks or classes using foundational biology models, students were not familiar with the models as presented in this study. Therefore, students needed to draw on their knowledge and understanding of these biological systems, as opposed to visual recognition of the model, to determine whether each model included an error (see supplementary Fig. 4 for an example of a presented model).

Students were screened to ensure that none had a learning disability, Attention-Deficit/Hyperactivity Disorder, experience of concussion, or other neurological diagnosis that might impact neural response patterns and that none had contraindications to MRI. One student was excluded from analyses because they consistently gave the same response to every task trial. Of the final analytic sample (Valid *N* = 50, *M*_age_ = 19.62, SD_age_ = .0.90), 35 (70%) were first-year freshmen, 12 (24%) sophomores, and three (6%) juniors. Seven (14%) were first-generation college students. Forty-three (86%) were European American/White, three (6%) Hispanic, three (6%) Asian, and one (2%) identified as both European American/White and Hispanic. All but two students were native English speakers, 38 (76%) were female, 10 (20%) male, and two (4%) another gender. The average grade-point average (GPA), on a 4.0 scale and collected through a third party from the Registrar’s Office, was 3.54 (SD = 0.53). The average final course grade from the introductory life sciences course, also on a 4.0 scale, was 3.55 (SD = 0.62). For analyses, final course grades are used, rather than cumulative GPAs, because final course grades are a more concise measure of student biology knowledge. On average, participants’ combined aggregated standardized score on the Kaufman Brief Intelligence Test, Second Edition (KBIT-2)^88^ was 103.37 (SD = 11.07), with all students falling within two standard deviations of the normative mean.

### Procedure

All procedures were approved by the University of Nebraska Human Subjects Protection Committee, and participants provided written informed consent to participation. Scans were also sent to a radiologist for review and students were informed of any incidental findings, with all of these being limited to sinus congestion. Students were compensated with $50 cash after attending a 2-h appointment at the university’s imaging center, where they completed the fMRI task. Study appointments occurred after the semester’s fourth week to allow students to become familiar with core course concepts and with the process of evaluating models or diagrams in biology.

Students underwent MRI in a 3 T Siemens Skyra scanner using a 32-channel head coil. After being fitted with ear protection, students reclined on the scanning table. First, a T1-weighted MPRAGE was acquired (TR = 1, TE = 2.95 ms, voxel size = 1 mm^3^, flip angle = 9, field of view = 270,176 sagittal slices) for registration purposes. This scan was followed by T2*-weighted echoplanar images (TR = 1 s, TE = 25 ms, 3 mm voxels, flip angle = 90°, FOV = 224 mm) collected during the model evaluation task.

### Model evaluation task

**Fig. 6:**
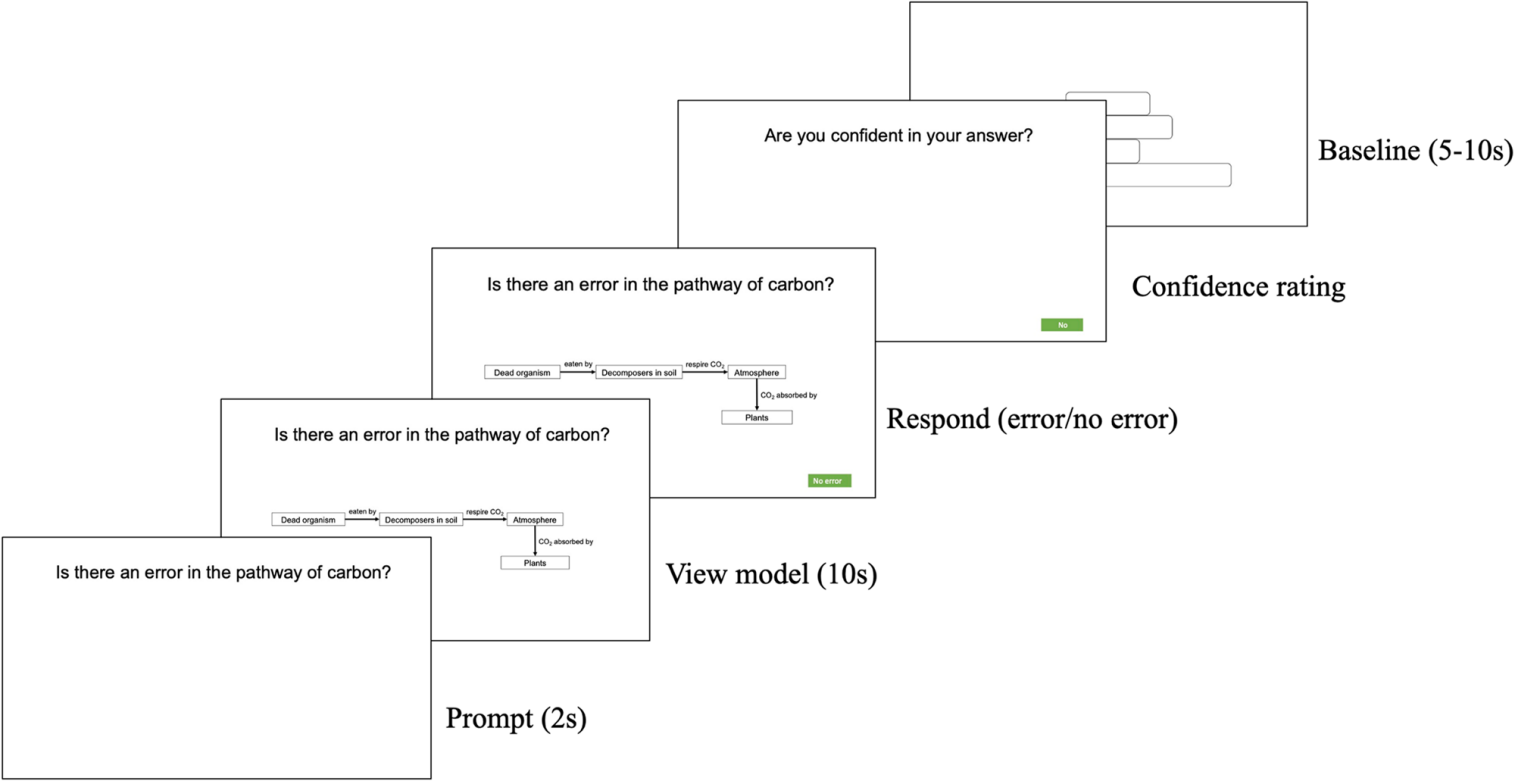
Model evaluation task students completed in the MRI scanner. The model evaluation task required participants to examine and determine whether the presented biology models contained errors, as well as to assess their confidence level for each model.

In the scanner, participants evaluated a series of models, formatted as flow charts, diagrams, or textbook-like images, that captured a breadth of content from the introduction to the life sciences course (e.g., human evolution, central dogma, genetic mutation). Stimuli were designed to examine the participants’ ability to detect errors and inhibit misconceptions (Fig. 6). To provide context for the model, students first saw a 2-s prompt, e.g., “Is there an error in the relationships?” They then viewed a model and the same prompt for 10 s, after which they could take up to 30 s to indicate via a response pad whether the model was correct or incorrect. Lastly, participants were cued to indicate whether they were confident in their response (yes or no). Participants completed three separate, randomly ordered runs lasting approximately 5 min each. Each run was composed of 12 randomly ordered trials. Generally, there were two incorrect versions for every correct version of a model, with 14 total correct models and 22 error-containing trials. Trials were followed by a baseline rest period with a jittered interval of 2–10 s, during which students saw random figures extracted from the model stimuli but without any words. Because students took varying times to respond, the number of volumes collected for each student varied between 231 and 424 volumes per run.

### Data analysis

Collecting multiple confidence ratings affords calculation of a simple phi coefficient (ϕ), the correlation between students’ accuracy and confidence ratings across all trials of a task^65^. Although widely used in the education literature, this method is susceptible to bias based on the participant’s task performance and does not adequately parse students’ confidence bias—their general tendency to make high or low confidence ratings—from their capacity to discriminate correct from inaccurate responses^35,70^. Consequently, cognitive neuroscience researchers recommend using model-based Signal Detection Theory metrics to provide response-bias-free measures of how precisely confidence ratings track task accuracy^64,65^. These measures include *meta-d’*, a measure of Student’s metacognition that is conditioned on their task performance distribution, and *metacognitive efficiency*, which reflects the difference between a student’s sensitivity to their performance and their actual task performance. An ‘ideal metacognitive observer’ should exhibit little difference between their meta-*d* and their task performance^65^. Such signal detection metrics parse students’ confidence biases and task performance from their capacity to discriminate optimally from less optimal performance, enabling a deeper understanding of the implications of different aspects of metacognition for learning.

Using SPSS^89^, we calculated our primary metric for calibration accuracy, the phi coefficient (ϕ), which reflects the correlation between a student’s accuracy and confidence scores. Two students were excluded from this analysis because they reported that they were confident on every trial; therefore, we could not calculate their ϕ. We also calculated students’ total confidence (i.e., the total number of times they responded ‘yes’ to the confidence prompt) and total accuracy across all trials and runs of the task, as well as creating a biased score based on the proportion of inaccurate trials where students reported they were confident—the proportion of accurate trials on which they were confident^89^. We used MATLAB scripts developed by Fleming^64^ to calculate a type 1 *d*’ score, a type 2 meta-*d*’ score, and a metacognitive efficiency score based on Bayesian estimation using Markov Chain Monte Carlo simulation implemented with the JAGS package. The advantage of this Bayesian approach to estimating these metacognitive metrics is that it is more robust with small trial numbers and can be used even when individuals have 0 responses in a particular cell. We used *d*’—meta *d*’ as our estimate of metacognitive efficiency because the alternative, ratio-based metacognitive efficiency score resulted in some extreme values.

Functional MRI data were processed and analyzed using the FMRIB Software Library^90^. Images were skull stripped, high pass filtered at 0.01 Hz, realigned, spatially smoothed with a 5 mm Gaussian kernel, registered to the T1 image using boundary-based registration, and normalized to the MNI 2 mm template. In the first level models for each run, we regressed the fMRI signal on task onsets convolved with a double gamma hemodynamic response function. The interval of focus was between when participants viewed the model and prompt to when they responded whether the model was correct or incorrect, i.e., the ‘view model’ and ‘respond’ phases in Fig. 1. The ‘confidence’ and prompt slides were treated as nuisance regressors in the design matrix, along with regressors for motion and temporal derivatives. Contrasts were performed for trials reflecting Error > No Error models. Furthermore, we contrasted trials where students responded they were Confident > Not confident and trials where they were Confident and Accurate > Confident and Inaccurate in their responses. Parameters for each participant were averaged in fixed effects models and passed to a group mixed effects analysis. In separate models, we added students’ metacognitive scores as covariates of interest in the design matrix for the Error > No error contrast. All contrasts were performed with a *Z* threshold of 3.1, *p* < 0.001 and a cluster-corrected *p* < 0.05.

### Reporting summary

Further information on research design is available in the Nature Research Reporting Summary linked to this article.

### Supplementary information

Supplemental Material

Reporting summary

## Data Availability

Group statistical maps are available on NeuroVault at https://neurovault.org/collections/13902/. Student behavioral data is available at the UNL data repository: 10.32873/unl.dr.20230419. Raw MRI data will be made available to researchers upon reasonable request and is not publicly available because participants did not agree to public data access during informed consent.

## Code availability

Analytic code is available from the authors upon request.
