# Supplementary material for: Relation of life sciences students’ metacognitive monitoring to neural activity during biology error detection
Source: NPJ Sci Learn. 2024 Mar 4;9:16. doi: 10.1038/s41539-024-00231-z (PMC10912288; doi:10.1038/s41539-024-00231-z)
Supplement: Supplementary file 1 — Supplemental Material [file 41539_2024_231_MOESM1_ESM.pdf]

Supplementary Materials

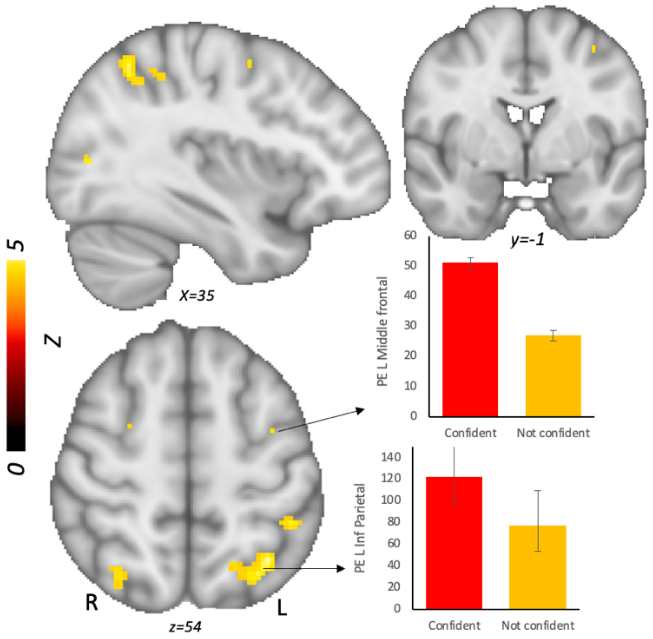

Supplementary figure 1: Confident > Not confident contrast with voxelwise height thresholding at $p < .05$. Error bars: Standard deviation.

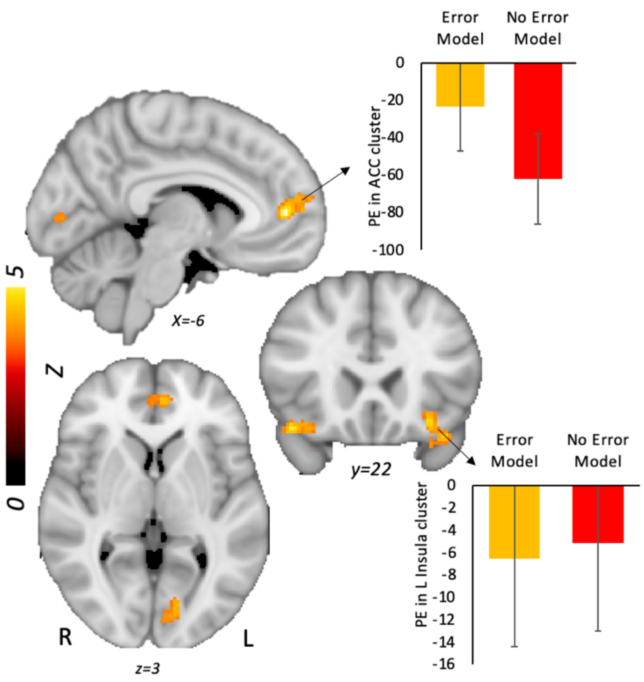

Supplementary figure 2: Error > No error models for trials where students responded they were confident. Error bars: Standard deviation.

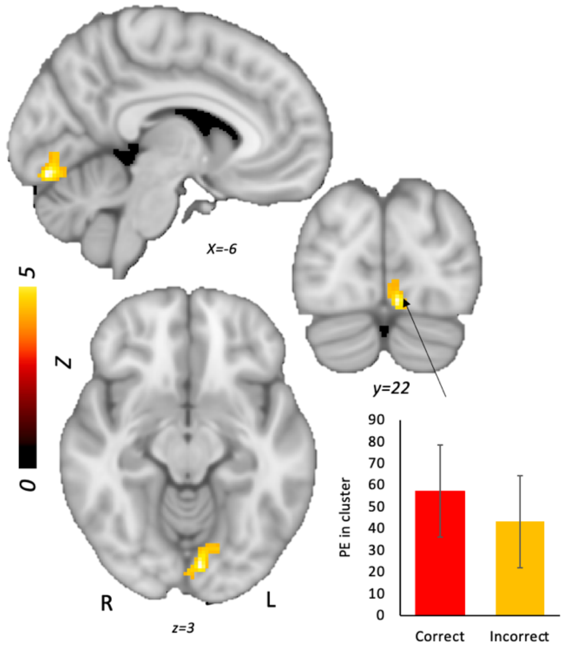

Supplementary figure 3: Correct > Incorrect responses for trials where students responded that they were confident. Error bars: Standard deviation.

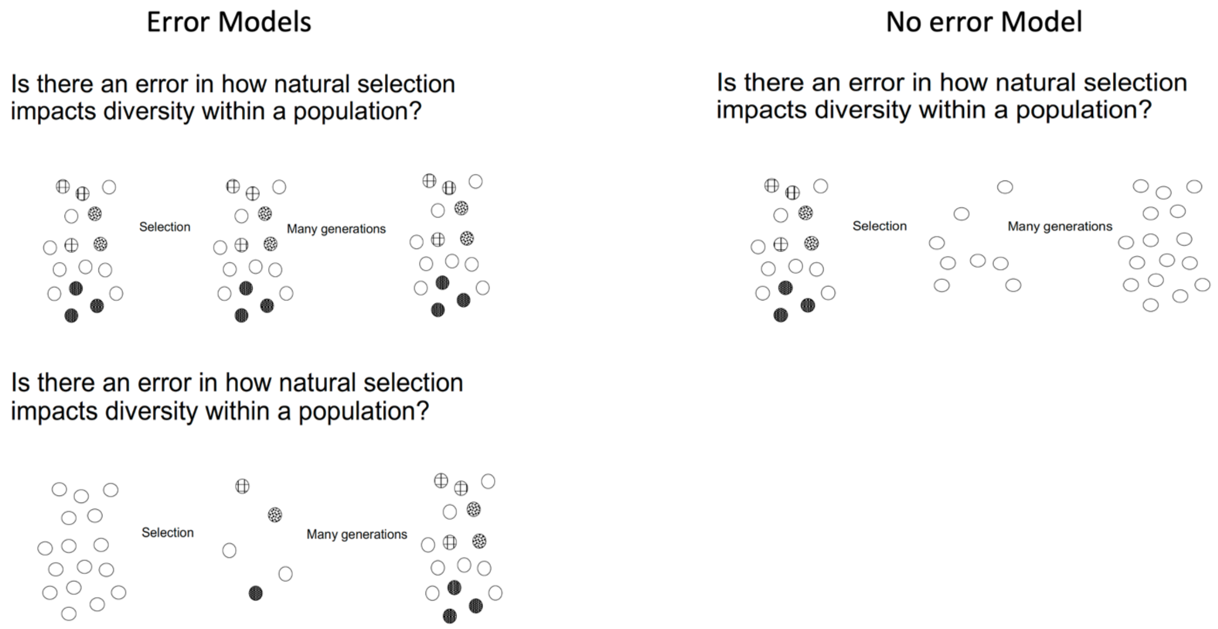

Supplementary figure 4: Example of models presented in scanner. Students must understand how mutations increase genetic variation within a population to evaluate whether the model is correct.

Please view https://identifiers.org/neurovault.collection:13902 more information.
Corresponding findings are listed below:

- https://identifiers.org/neurovault.image:796396 (Figure 2)
- https://identifiers.org/neurovault.image:796399 (Figure 3)
- https://identifiers.org/neurovault.image:796400 (Figure 4)
- https://identifiers.org/neurovault.image:796401 (Figure 4)
- https://identifiers.org/neurovault.image:796402 (Figure 5)
- https://identifiers.org/neurovault.image:796397 (Supplementary figure 1
- https://identifiers.org/neurovault.image:796398 (Supplementary figure 2)
