# Supplementary material for: Relation of life sciences students’ metacognitive monitoring to neural activity during biology error detection
Source: NPJ Sci Learn. 2024 Mar 4;9:16. doi: 10.1038/s41539-024-00231-z (PMC10912288; doi:10.1038/s41539-024-00231-z)
Supplement: Supplementary file 2 — Reporting summary [file 41539_2024_231_MOESM2_ESM.pdf]

nature portfolio

Corresponding author(s): Mei Grace Behrendt

Last updated by author(s): 25 September 2023



# Reporting Summary

Nature Portfolio wishes to improve the reproducibility of the work that we publish. This form provides structure for consistency and transparency in reporting. For further information on Nature Portfolio policies, see our Editorial Policies and the Editorial Policy Checklist.

Please do not complete any field with "not applicable" or n/a. Refer to the help text for what text to use if an item is not relevant to your study.
For final submission: please carefully check your responses for accuracy; you will not be able to make changes later.

## Statistics

For all statistical analyses, confirm that the following items are present in the figure legend, table legend, main text, or Methods section.

| n/a | Confirmed | |
|---|---|---|
| ☐ | ☑ | The exact sample size ($n$) for each experimental group/condition, given as a discrete number and unit of measurement |
| ☐ | ☑ | A statement on whether measurements were taken from distinct samples or whether the same sample was measured repeatedly |
| ☐ | ☑ | The statistical test(s) used AND whether they are one- or two-sided<br>*Only common tests should be described solely by name; describe more complex techniques in the Methods section.* |
| ☐ | ☑ | A description of all covariates tested |
| ☐ | ☑ | A description of any assumptions or corrections, such as tests of normality and adjustment for multiple comparisons |
| ☐ | ☑ | A full description of the statistical parameters including central tendency (e.g. means) or other basic estimates (e.g. regression coefficient) AND variation (e.g. standard deviation) or associated estimates of uncertainty (e.g. confidence intervals) |
| ☐ | ☑ | For null hypothesis testing, the test statistic (e.g. $F$, $t$, $r$) with confidence intervals, effect sizes, degrees of freedom and $P$ value noted<br>*Give P values as exact values whenever suitable.* |
| ☐ | ☑ | For Bayesian analysis, information on the choice of priors and Markov chain Monte Carlo settings |
| na | ☐ | For hierarchical and complex designs, identification of the appropriate level for tests and full reporting of outcomes |
| ☐ | ☑ | Estimates of effect sizes (e.g. Cohen's $d$, Pearson's $r$), indicating how they were calculated |

*Our web collection on statistics for biologists contains articles on many of the points above.*

## Software and code

Policy information about availability of computer code

| | |
|---|---|
| Data collection | MRI in a 3 Tesla Siemens Skyra scanner and Psychopy |
| Data analysis | IBM SPSS, FMRIB Software Library, and MATLAB scripts |

For manuscripts utilizing custom algorithms or software that are central to the research but not yet described in published literature, software must be made available to editors and reviewers. We strongly encourage code deposition in a community repository (e.g. GitHub). See the Nature Portfolio guidelines for submitting code & software for further information.

## Data

Policy information about availability of data

All manuscripts must include a data availability statement. This statement should provide the following information, where applicable:

- Accession codes, unique identifiers, or web links for publicly available datasets
- A description of any restrictions on data availability
- For clinical datasets or third party data, please ensure that the statement adheres to our policy

Statistical maps are available on NeuroVault at https://neurovault.org/collections/13902/ and in the Supplementary Information.
Student behavioral data is available at the UNL data repository: https://doi.org/10.32873/unl.dr.20230419.

## Research involving human participants, their data, or biological material

Policy information about studies with human participants or human data. See also policy information about sex, gender (identity/presentation), and sexual orientation and race, ethnicity and racism.

| | |
|---|---|
| Reporting on sex and gender | 38 (76%) were female, ten (20%) male, and two (4%) another gender |
| Reporting on race, ethnicity, or other socially relevant groupings | Forty-three (86%) were European American/White, three (6%) Hispanic, three (6%) Asian, and one (2%) identified as both European American/White and Hispanic. |
| Population characteristics | 35 (70%) were first-year freshmen, 12 (24%) sophomores, and three (6%) juniors. Seven (14%) were first-generation college students. |
| Recruitment | Fifty-one undergraduate students were recruited through class announcements from five separate sections of one introductory life sciences course\ taught by two different instructors in two consecutive academic years at a large Midwestern university. |
| Ethics oversight | All procedures were approved by the University of Nebraska Human Subjects Protection Committee, and participants provided written informed consent to participation. |

Note that full information on the approval of the study protocol must also be provided in the manuscript.

# Field-specific reporting

Please select the one below that is the best fit for your research. If you are not sure, read the appropriate sections before making your selection.

☐ Life sciences ☑ Behavioural & social sciences ☐ Ecological, evolutionary & environmental sciences

For a reference copy of the document with all sections, see nature.com/documents/nr-reporting-summary-flat.pdf

# Life sciences study design

All studies must disclose on these points even when the disclosure is negative.

| | |
|---|---|
| Sample size | |
| Data exclusions | |
| Replication | |
| Randomization | |
| Blinding | |

# Behavioural & social sciences study design

All studies must disclose on these points even when the disclosure is negative.

| | |
|---|---|
| Study description | This study aimed to determine how variation in undergraduate life sciences students' metacognitive calibration relates to their brain activity when evaluating the accuracy of biological models. |
| Research sample | Fifty-one undergraduate students |
| Sampling strategy | Fifty-one undergraduate students were recruited through class announcements from five separate sections of one introductory life sciences course taught by two different instructors in two consecutive academic years at a large Midwestern university. |
| Data collection | Participants evaluated a series of biology models in a MRI to examine their ability to detect errors and inhibit misconceptions |
| Timing | First assessment of a longitudinal study |
| Data exclusions | One student was excluded from analyses because they consistently gave the same response to every task trial. |
| Non-participation | NA |
| Randomization | NA |

# Ecological, evolutionary & environmental sciences study design

All studies must disclose on these points even when the disclosure is negative.

Study description

Research sample

Sampling strategy

Data collection

Timing and spatial scale

Data exclusions

Reproducibility

Randomization

Blinding

Did the study involve field work? ☐ Yes ☐ No

## Field work, collection and transport

Field conditions

Location

Access & import/export

Participants e a series of biology models in a MRI scanner and then completed some cognitive assessments.

Disturbance

# Reporting for specific materials, systems and methods

We require information from authors about some types of materials, experimental systems and methods used in many studies. Here, indicate whether each material, system or method listed is relevant to your study. If you are not sure if a list item applies to your research, read the appropriate section before selecting a response.

## Materials & experimental systems

| n/a | Involved in the study |
|---|---|
| ☐ | ☐ Antibodies |
| ☐ | ☐ Eukaryotic cell lines |
| ☐ | ☐ Palaeontology and archaeology |
| ☐ | ☐ Animals and other organisms |
| ☐ | ☐ Clinical data |
| ☐ | ☐ Dual use research of concern |
| ☐ | ☐ Plants |

## Methods

| n/a | Involved in the study |
|---|---|
| ☐ | ☐ ChIP-seq |
| ☐ | ☐ Flow cytometry |
| ☐ | ☐ MRI-based neuroimaging |

## Antibodies

Antibodies used

Validation

## Eukaryotic cell lines

Policy information about cell lines and Sex and Gender in Research

Cell line source(s)

Authentication

Mycoplasma contamination

Commonly misidentified lines
(See ICLAC register)

## Palaeontology and Archaeology

Specimen provenance

Specimen deposition

Dating methods

☐ Tick this box to confirm that the raw and calibrated dates are available in the paper or in Supplementary Information.

Ethics oversight

Note that full information on the approval of the study protocol must also be provided in the manuscript.

## Animals and other research organisms

Policy information about studies involving animals; ARRIVE guidelines recommended for reporting animal research, and Sex and Gender in Research

Laboratory animals

Wild animals

Reporting on sex

Field-collected samples

Ethics oversight

Note that full information on the approval of the study protocol must also be provided in the manuscript.

## Clinical data

Policy information about clinical studies

All manuscripts should comply with the ICMJE guidelines for publication of clinical research and a completed CONSORT checklist must be included with all submissions.

Clinical trial registration

Study protocol

Data collection

Outcomes

## Dual use research of concern

Policy information about dual use research of concern

### Hazards

Could the accidental, deliberate or reckless misuse of agents or technologies generated in the work, or the application of information presented in the manuscript, pose a threat to:

| No | Yes | |
|---|---|---|
| ☐ | ☐ | Public health |
| ☐ | ☐ | National security |
| ☐ | ☐ | Crops and/or livestock |
| ☐ | ☐ | Ecosystems |
| ☐ | ☐ | Any other significant area |

### Experiments of concern

Does the work involve any of these experiments of concern:

| No | Yes | |
|---|---|---|
| ☐ | ☐ | Demonstrate how to render a vaccine ineffective |
| ☐ | ☐ | Confer resistance to therapeutically useful antibiotics or antiviral agents |
| ☐ | ☐ | Enhance the virulence of a pathogen or render a nonpathogen virulent |
| ☐ | ☐ | Increase transmissibility of a pathogen |
| ☐ | ☐ | Alter the host range of a pathogen |
| ☐ | ☐ | Enable evasion of diagnostic/detection modalities |
| ☐ | ☐ | Enable the weaponization of a biological agent or toxin |
| ☐ | ☐ | Any other potentially harmful combination of experiments and agents |

## Plants

Seed stocks

Novel plant genotypes

Authentication

## ChIP-seq

### Data deposition

☐ Confirm that both raw and final processed data have been deposited in a public database such as GEO.

☐ Confirm that you have deposited or provided access to graph files (e.g. BED files) for the called peaks.

Data access links
*May remain private before publication.*

Files in database submission

Genome browser session
(e.g. UCSC)

### Methodology

Replicates

Sequencing depth

Antibodies

Peak calling parameters

Data quality

Software

## Flow Cytometry

### Plots

Confirm that:

- [ ] The axis labels state the marker and fluorochrome used (e.g. CD4-FITC).
- [ ] The axis scales are clearly visible. Include numbers along axes only for bottom left plot of group (a 'group' is an analysis of identical markers).
- [ ] All plots are contour plots with outliers or pseudocolor plots.
- [ ] A numerical value for number of cells or percentage (with statistics) is provided.

### Methodology

| | |
|---|---|
| Sample preparation | |
| Instrument | |
| Software | |
| Cell population abundance | |
| Gating strategy | |

- [ ] Tick this box to confirm that a figure exemplifying the gating strategy is provided in the Supplementary Information.

## Magnetic resonance imaging

### Experimental design

| | |
|---|---|
| Design type | Slow event design |
| Design specifications | Participants evaluated a series of biology models in a MRI scanner to examine the participants' ability to detect errors and inhibit misconceptions. |
| Behavioral performance measures | Meta d' and Phi Coeffcient |
| Imaging type(s) | T1, T2* |
| Field strength | 3 Tesla Siemens Skyra scanner using a 32-channel head coil |
| Sequence & imaging parameters | |
| Area of acquisition | Whole brain |

Diffusion MRI  [ ] Used  [x] Not used

### Preprocessing

| | |
|---|---|
| Preprocessing software | FMRIB Software Library |
| Normalization | Normalized to the MNI 2mm template |
| Normalization template | Normalized to the MNI 2mm template |
| Noise and artifact removal | Included motion regression in analyses |
| Volume censoring | 231 and 424 volumes per run |

### Statistical modeling & inference

| | |
|---|---|
| Model type and settings | General linear model |
| Effect(s) tested | Correlations; confident vs. not confident trials; calibrated vs. non calibrated |

Specify type of analysis: [x] Whole brain  [ ] ROI-based  [ ] Both

| | |
|---|---|
| Statistic type for inference (See Eklund et al. 2016) | Cluster thersholding at Z=3.1 |
| Correction | Random Field Theory Based Cluster Correction |

## Models & analysis

| n/a | Involved in the study |
|---|---|
| na | ☐ Functional and/or effective connectivity |
| na | ☐ Graph analysis |
| na | ☐ Multivariate modeling or predictive analysis |

| | |
|---|---|
| Functional and/or effective connectivity | None |
| Graph analysis | None |
| Multivariate modeling and predictive analysis | None |

This checklist template is licensed under a Creative Commons Attribution 4.0 International License, which permits use, sharing, adaptation, distribution and reproduction in any medium or format, as long as you give appropriate credit to the original author(s) and the source, provide a link to the Creative Commons license, and indicate if changes were made. The images or other third party material in this article are included in the article's Creative Commons license, unless indicated otherwise in a credit line to the material. If material is not included in the article's Creative Commons license and your intended use is not permitted by statutory regulation or exceeds the permitted use, you will need to obtain permission directly from the copyright holder. To view a copy of this license, visit http://creativecommons.org/licenses/by/4.0/
